# On the relation between grammatical number and cardinal numbers in development

**DOI:** 10.3389/fpsyg.2014.01132

**Published:** 2014-10-09

**Authors:** Barbara W. Sarnecka

**Affiliations:** Department of Cognitive Sciences, University of California at Irvine, Irvine, CA, USA

**Keywords:** cardinal, counting, language development, number, plural, grammatical number

## Abstract

This mini-review focuses on the question of how the grammatical number system of a child’s language may help the child learn the meanings of cardinal number words (e.g., “one” and “two”). Evidence from young children learning English, Russian, Japanese, Mandarin, Slovenian, or Saudi Arabic suggests that trajectories of number-word learning differ for children learning different languages. Children learning English, which distinguishes between singular and plural, seem to learn the meaning of the cardinal number “one” earlier than children learning Japanese or Mandarin, which have very little singular/plural marking. Similarly, children whose languages have a singular/dual/plural system (Slovenian and Saudi Arabic) learn the meaning of “two” earlier than English-speaking children. This relation between grammatical and cardinal number may shed light on how humans acquire cardinal-number concepts. There is an ongoing debate about whether mental symbols for small cardinalities (concepts for “oneness,” “twoness,” etc.) are innate or learned. Although an effect of grammatical number on number-word learning does not rule out nativist accounts, it seems more consistent with constructivist accounts, which portray the number-learning process as one that requires significant conceptual change.

There are different ways to convey numerical information in language. Suppose you and I meet for the first time, and you wonder whether I have children. (Of course you are too polite to ask.) During our conversation, I say, “I thought that as a developmental psychologist, I would find it easy to be a parent, but I don’t.” Now you know that I have at least one child. If I say, “I came to this conference to get away from my kids,” you know that I have two or more children, because the English word *kids* is plural, and must refer to sets of two or more. Finally, if I say, “My kids can’t stop arguing; they both want the last word,” you know that I have exactly two children, because the English word *both* always refers to sets of exactly two. (A rare example of dual marking in English.) Alternatively, you might simply ask whether I have children, and I might say, “Yes. I have two boys.”

As this example demonstrates, numerical information can be communicated via cardinal number words (“one,” “two,” “three,” etc.), but it can also be communicated via grammatical morphology, such as the *s* on the English word *kids*. English is a singular/plural language, meaning that it marks the difference between sets of one and sets of two or more. But not all languages do this. Numeral classifier languages such as Japanese and Mandarin have very little singular/plural marking (Downing, 1996). In these languages, saying “I have kid(s)” is like saying in English, “I am a parent.” It conveys no information at all about *how many* kids you have. Still other languages have singular/dual/plural marking systems, which pick out sets of one, sets of two, and sets of three or more. In these languages, dual-marked noun phrases refer to sets of exactly two, similar to the English word *both*. A few languages go even further, marking singular/dual/trial/plural for sets of one, two, three, and four or more, respectively, or marking singular/dual/paucal/plural where paucal marking picks out small sets (something like the English phrase “a handful”) and plural marking picks out larger sets (Corbett, 2000).

This mini-review focuses on the question of how of these two systems (grammatical number and cardinal numbers) may be related in development. There is some evidence that the grammatical number marking system of the language a child is learning may influence that child’s learning of the cardinal number system. Because cardinal number systems are functionally identical across languages while grammatical number systems differ, we can look at differences in children’s learning of cardinal numbers, and see if that learning bears the signatures of particular languages’ grammatical number systems.

When we do this, we find evidence that indeed, a language’s grammatical number system does seem to influence children’s learning of cardinal number words in that language. Children learning a language as English, which pervasively marks singular/plural, seem to learn the meaning of the number “one” earlier than children whose languages do not mark singular/plural, such as Japanese (Sarnecka et al., 2007). Similarly, children whose languages have a singular/dual/plural system (Slovenian and Saudi Arabic) appear to learn the meaning of “two” earlier than English-speaking children (Almoammer et al., 2013).

This is interesting, not because it tells us anything about how adult number concepts in any language, but because it may shed some light on how number concepts are acquired. There is an ongoing debate about whether mental symbols for small cardinalities (concepts for oneness, twoness, threeness, and the like) are innate or learned. Some proposals argue that these concepts are innate and shared with other animals (e.g., Gelman and Gallistel, 1978, 2004; Gelman and Butterworth, 2005; Butterworth et al., 2008). On these accounts, the challenge for the child learning language may just be to identify the words (i.e., cardinal number words) that match her innate concepts of oneness, twoness, threeness, etc.

On the other side of the debate, it is argued that humans are not born with concepts of oneness, twoness, threeness, etc., but must construct them (Le Corre and Carey, 2007; Carey, 2009). People in numerate societies construct these concepts during early childhood, in the course of learning the meanings for the cardinal number words “one,” “two,” “three,” and eventually the properties of the cardinal number system: that each number has a successor, that all sets of the same number can be put into one-to-one correspondence with each other, etc. (Izard et al., 2008, 2014; Sarnecka and Carey, 2008; Carey, 2009; Sarnecka and Wright, 2013; Sarnecka et al., in press).

## THE QUESTION

The question of how grammatical number might be related to cardinal number began with an observation about trajectories of number-word learning in English. In the early 1990s, Wynn (1990, 1992) first reported that children learn the meanings of cardinal number words one at a time and in order. Wynn showed this using the “Give-N” or “Give-a-number” task, in which she asked children to give her a certain number of items (e.g., “Give me one fish”; “Give me three fish,” etc.). She found that children’s performance moved through a predictable series of levels.

At the earliest (“pre-number-knower”) level, children do not distinguish among the different number words. Pre-number knowers might give one object for every number requested, or they might give a handful of objects for every number, but they show no sign of knowing the exact meaning of any number word. At the next level (called the “one-knower” level), children know that “one” means 1. On the Give-N task, one-knowers give exactly one object when asked for “one,” and they give two or more objects when asked for any other number. After this comes the “two-knower” level, where children give one object for “one,” and two objects for “two,” but do not reliably produce larger sets. This is followed by a “three-knower” level and (although Wynn didn’t find it because she never asked children for four objects) a “four-knower” level. After the four-knower level, children seem to learn the meanings of the higher cardinal number words in a different way-inferring their meanings from their place in the counting list rather than learning them individually as they did with the small numbers (Carey, 2009). Children who have done this (i.e., who have figured out how the counting system represents cardinal numbers) are called “Cardinal-principle knowers.”

The age at which children master these knower levels differs from one child to another, but in the most commonly studied population (English-speaking children from relatively privileged socioeconomic backgrounds), children typically reach the “one-knower” level some time during their second or third year (i.e., between 24 and 47 months old) and reach the final, “cardinal-principle-knower” level about 1 year later, between about 34 and 51 months (Sarnecka et al., in press).

As a graduate student reading Wynn’s work in the late 1990s, I noticed a parallel between children’s number-word learning and grammatical number systems. Both follow a rigid hierarchy: a child who understands “two” always understands “one” as well, just as a language that marks dual always marks singular as well. There do not seem to be children who understand “three” but *not* “one” and “two,” just as there are no languages that grammatically mark trial but *not* singular and dual. In a way, pre-number-knowers are like speakers of numeral classifier languages (e.g., Japanese); one-knowers are like speakers of singular/plural languages (e.g., English); and two-knowers were like speakers of singular/dual/plural languages (e.g., Slovenian).

A striking feature of number-word learning in English is the really long one-knower level. Wynn (1992) reported that children seemed to spend many months at the one-knower level-much longer than they spent as two-knowers or three-knowers. Why should that be the case? One possible explanation is that because English is a singular/plural language, English-speaking children must pay special attention to the distinction between one and other set sizes. English-speaking children show understanding of singular/plural marking between 20 and 24 months of age (Kouider et al., 2006); it is possible that this knowledge helps children learn the meaning of “one” sooner than they would if their language did not distinguish singular from plural. This explanation can be tested by comparing number-word learning in English to number-word learning in Japanese, which generally does not distinguish singular from plural.

A different possibility is that “one” is learned earlier than “two” simply because “one” is much more frequent in everyday speech. Across languages, “one” is more frequent than “two”; “two” is more frequent than “three,” and so on (Dehaene and Mehler, 1992). The frequency of “one” is particularly high in English, where it appears not only in counting, but also in deictic and anaphoric contexts (e.g., “Look at that one” or, “I’m making sandwiches-do you want one?”) This explanation can be tested by comparing English-speaking children’s number-word learning to that of children speaking Russian, a singular/plural language where the cardinal number “one” does not appear in non-numeric contexts.

## THE EVIDENCE

My collaborators and I administered Wynn’s Give-a-number task, as well as a counting task, to young children living in Ann Arbor, MI, USA; St. Petersburg, Russia, and Kobe, Japan (Sarnecka et al., 2007). Children in each group ranged in age from 2 years, 9 months to 3 years, 6 months, and the mean age for each group was 3 years, 2 months.

We found that more English- and Russian-speakers knew the meaning of “one” than did their Japanese counterparts, supporting the idea that speaking a language with singular/plural marking helps children learn the meaning of “one.” Comparing English to Russian, we found that Russian-speakers were actually more likely to know “one” than English speakers, even though the Russian word for “one” appears less frequently in everyday speech than the English word “one.” Thus, the data did not support the idea that the overall high frequency of “one” relative to other numbers causes English-speaking children to reach the one-knower level sooner. Rather, it seems to be the presence of singular/plural marking in the language that makes the difference.

One question that arose about these findings was whether Japanese was the best choice to represent non-singular/plural marking languages. Number-word learning in Japanese is potentially complicated by the presence of two count lists, which sound nothing at all alike. (One of the lists begins *ichi*, *ni*, *san*, *shi*, *go*… the other begins *hitotsu*, *futatsu*, *mitsu*, *yotsu*, *itsutsu*…) Both of the lists are commonly used for numbers up to 10 (although only the *ichi*, *ni*, *san* list is used for numbers above 10), so it is reasonable to ask whether Japanese children might take longer to learn the number-word meanings, just because the input they receive for each number is potentially divided between two different word forms.

We addressed this question in the 2007 paper by arguing that Russian-speaking children also have to deal with different word forms, as numbers are declined for gender and case. For example, the word *one* in Russian may take any of the following forms: *odin*, *odna*, *odno*, *odni*, *odnu*, *odnovo*, *odnikh*, *odnoy*, *odnom*, *odnomu*, *odnim*, *odnimi*. But this argument is not wholly convincing, first because these forms of *one* are not as different from each other as *hitotsu* and *ichi*, and second because when people actually count in Russian, the number words are usually in the nominative case, so the count list sounds the same every time. Japanese, on the other hand, actually has two different counting lists, which could be a serious confound. So it is important to note that the finding of children learning “one” later in a non-singular/plural language has not only been replicated in Japanese (Barner et al., 2009b) but is also found in Mandarin, which very sensibly has only one count list (Li et al., 2003).

Further evidence for a link between grammatical number and cardinal number-word learning has recently come from a study with young speakers of two languages with singular/dual/plural systems: Slovenian and Saudi Arabic (Almoammer et al., 2013). The study tested 2- to 4-year-old children in Slovenian, and 3- and 4-year-old children in Arabic. Significantly more children knew the meaning of “two” in the dual-marking languages than in age-matched groups of English speakers. Slovenian children learned “two” sooner than English-speaking children despite not being able to count as well as the English speakers, which is surprising because counting ability would seem to indicate experience with numbers. (No counting data were available for the Saudi Arabic-speaking children.) In both Slovenian and Saudi Arabic, children’s understanding of the grammatical dual forms was correlated with their knowledge of the cardinal number “two.”

Moreover, just as English-speaking children seem to spend a long time at the one-knower level, so do Slovenian-speaking children spend a long time at the two-knower level. Although they learn “two” earlier, they stay at the two-knower level for longer, taking more time to learn “three” and higher numbers than children in the other language environments studied. This connection between grammatical dual marking and learning “two” is interesting because it shows that the meaning of “two” doesn’t follow automatically from “one,” but requires additional inference, for which dual-marking languages provide additional evidence. This pattern is consistent with Carey’s (2009) account, in which the meanings of “one” through “four” are learned individually, whereas the meanings of the higher numbers are learned as a group, when the child comes to understand the cardinal principle.

At least one qualification to these findings should be noted. In our original paper, we speculated that children learning singular/plural languages like English may initially understand “one” as meaning *singular* as opposed to *plural* (Sarnecka et al., 2007). As an example, we suggested that children may treat “one” like the indefinite article “a(n).” (In fact, the number “one” and the indefinite article were originally the same word in English, as they are today in languages such as Spanish and French.)

However, one study compared English-speaking children’s use of “one” and “a(n),” and found that children sometimes treat them differently. Children were shown a plate with two apples on it, and were asked either, “Is there *an apple* on the plate?” or “Is there *one apple* on the plate?” (Barner et al., 2009a). Children generally agreed with the statement that there was “an apple” on the plate, but disagreed with the statement that there was “one apple,” indicating that they treated the number “one” as upper-bounded (i.e., more than one is not one), but did not treat the word “a(n)” that way. Thus, although grammatical number helps children learn the meaning of “one,” they do not treat the words as identical.

## CONCLUSION

It does appear that the child’s learning of cardinal numbers is affected by the grammatical number system of his or her native language. Children whose languages mark singular/plural learn the cardinal meaning of the counting word “one” sooner than children whose languages do not mark the singular/plural distinction. Similarly, children whose languages distinguish dual from both singular and plural seem to learn “two” earlier than children in other language environments.

Even more interesting, perhaps, is the slight delay that children seem to experience in learning the first number *not* grammatically marked by their language. That is, children speaking singular/plural languages not only learn “one” a little sooner, but also seem to stay at the one-knower stage a bit later than children speaking other languages. Similarly, children whose languages include dual marking not only learn “two” earlier, but also seem to linger at the two-knower level longer than children in other language environments.

This suggests that the process of learning numbers that are grammatically marked (i.e., “one” for speakers of singular/plural languages; “one” and “two” for speakers of singular/dual/plural languages) may differ from the process of learning numbers that are not so marked. Children may use different sources of information to learn the meanings of grammatically marked vs. unmarked numbers. When the information from grammar runs out (e.g., when English speakers move on to learning “two” or Slovenian speakers to learning “three”), children must rely on some other source of information to figure out the next number word. This results in a slight delay in learning, relative to speakers of languages such as Japanese where all numbers are learned without the help of grammatical number marking^1^.

If number-word learning is affected by the child’s language environment, what if anything does that tell us about the innateness of number concepts? On balance, this evidence seems most compatible with constructivist views, because it implies that number-word learning requires significant conceptual change.

When a child’s language environment highlights certain numerical distinctions (i.e., one/more than one, or one/two/more than two), these distinctions become more salient to the child, and therefore more available as candidate meanings for counting words, speeding the number-acquisition process. Perhaps having to distinguish between individuals and sets (or between individuals, pairs, and larger sets), speeds number learning by making concepts such as *individual, pair,* and *set* available as candidate meanings for cardinal number words.

Similarly, children slow down a bit when they encounter the first number whose meaning is not grammatically marked. This implies that children learn grammatically marked and unmarked numbers by different processes, which is also seems more consistent with a constructivist than a nativist framework.

Of course, it is possible to hold a nativist position and still allow that grammatical distinctions can help children map counting words to innate number concepts. But overall, these effects of environment on learning seem to support constructivist accounts, where children build concepts of oneness, twoness, threeness, etc. based on the particular evidence they have available. When the grammatical number system of a language highlights different numerical distinctions, trajectories of cardinal number learning differ in systematic and predictable ways. This implies that becoming numerate involves something more than simply a matching a verbal counting list to an innate, non-verbal counting list. Numerate children, it implies, are made and not born.

### Conflict of Interest Statement

The author declares that the research was conducted in the absence of any commercial or financial relationships that could be construed as a potential conflict of interest.

## Footnotes

1 Thanks to Emily Mather for pointing out the potential significance of the delay in learning the first grammatically unmarked number.
